# Possibility Study of Scale Invariant Feature Transform (SIFT) Algorithm Application to Spine Magnetic Resonance Imaging

**DOI:** 10.1371/journal.pone.0153043

**Published:** 2016-04-11

**Authors:** Dong-Hoon Lee, Do-Wan Lee, Bong-Soo Han

**Affiliations:** 1 Division of MR Research, Department of Radiology, Johns Hopkins University School of Medicine, Baltimore, Maryland, United States of America; 2 Department of Radiological Science, College of Health Science, Yonsei University, Wonju, Rep. of Korea; Banner Alzheimer's Institute, UNITED STATES

## Abstract

The purpose of this study is an application of scale invariant feature transform (SIFT) algorithm to stitch the cervical-thoracic-lumbar (C-T-L) spine magnetic resonance (MR) images to provide a view of the entire spine in a single image. All MR images were acquired with fast spin echo (FSE) pulse sequence using two MR scanners (1.5 T and 3.0 T). The stitching procedures for each part of spine MR image were performed and implemented on a graphic user interface (GUI) configuration. Moreover, the stitching process is performed in two categories; manual point-to-point (mPTP) selection that performed by user specified corresponding matching points, and automated point-to-point (aPTP) selection that performed by SIFT algorithm. The stitched images using SIFT algorithm showed fine registered results and quantitatively acquired values also indicated little errors compared with commercially mounted stitching algorithm in MRI systems. Our study presented a preliminary validation of the SIFT algorithm application to MRI spine images, and the results indicated that the proposed approach can be performed well for the improvement of diagnosis. We believe that our approach can be helpful for the clinical application and extension of other medical imaging modalities for image stitching.

## Introduction

The vertebral column is an anatomical structure composed of cervical, thoracic and lumbar spine (C-T-L spine), and sacrum and coccyx. Diagnostic evaluation of these structures is important for assessing pathology and a crucial tool to achieve this goal is imaging, particularly magnetic resonance (MR) imaging. However, it is difficult to acquire the whole C-T-L spine image at once due to the limited field-of-view (FOV) of receiver coil system [1–3]. Thus, image stitching method that made a single image through the separate images was widely used to perform the combining images, effectively [3, 4]. Although using the multi-channel phased array C-T-L spine coil is enabled to acquire the C-T-L spine image at once, the use of image stitching method is necessary not only in C-T-L spine image and but also in whole-body MR image acquisition.

The image stitching methods are generally related with the image deformable registration methods because of the image stitching procedures based on the spatial-temporal registration application between two images. Moreover, automatic landmark detection and extraction process were basically performed to the image-based deformable registration. Harris and Stephens have shown the landmark detection results using corner detectors at a single scale [5]. Shi and Tomasi also have shown the method for corner detectors [6]. These approaches are very sensitive to changes in image scale, so it has some limitations in the accuracy and stability of feature detection. To overcome this problem, Lowe extended the local feature approach to achieve scale invariance [7, 8]. The invariance properties of feature extraction methods are crucial to detect points in temporal image series. Scale Invariant Features Transform (SIFT) algorithm proposed by Lowe have been used to generate the image features and to take local feature vectors [7, 8]. Each of these feature vectors is invariant to any translation, rotation, or scaling of the image. The SIFT algorithm has been used in application to two-dimensional images, and is expanded to application in multi-dimensional image by Cheung and Hamarneh [9].

In this study, we applied SIFT algorithm to C-T-L spine MR image for stitching of them to investigate the feasibility and reproducibility. Furthermore, this proposed SIFT algorithm to stitching of MR spine image is implemented on a graphic user interface (GUI) to ease to applications. For quantitative analysis, we compared the results between the use of SIFT algorithm and the use of commercial algorithm mounted in an MRI system. Due to the lack of the gold standard results for the imaging stitching, we have assumed two conditions to derive the comparison results in this study; i) the stitching results from the commercially mounted MRI system were considered as the main comparison target (ground truth). ii) the stitching algorithms in the commercial MRI system were provided relatively accurate stitching results in clinical fields because those results have already been used widely in clinical fields.

## Materials and Methods

### MRI data acquisition

Five male healthy subjects (mean age ± standard deviation: 26 ± 2.1 years) participated in this study. All subjects were provided written informed consent. All procedures and subjects were approved by Yonsei Univesity Wonju Sevrance Christian Hospital institutional review board. Each part of C-T-L spine images were acquired using two MRI system: 1.5 T MRI system (SM160, SCIMEDIX, South Korea) using Fast Spin Echo (FSE) sequence with following parameters (Time of Echo (TE) / Time of Repetition (TR) = 16 / 560 ms, Field of View (FOV) = 350 mm^2^, Echo Train Length (ETL) = 4, matrix size = 420 × 420, slice thickness = 4 mm and number of slices = 6) and Philips 3.0 T MRI system (Achieva 3.0 T; Philips Medical Systems, The Best, Netherlands) using FSE sequence with following parameters (TE / TR = 120 / 3,000 ms, FOV = 288 mm^2^, ETL = 27, matrix size = 576 × 576, slice thickness = 4.4 mm and number of slices = 5).

### Image stitching process

The implementation of image stitching method is performed in two categories; manual point-to-point (mPTP) selection and automated point-to-point (aPTP) selection. For the mPTP approach, the user specified corresponding matching points between two images (C-spine and T-spine images) at the overlapping area, and then image transformation process was performed based on the set of points. The second image stitching process was performed using the stitched image (C-T spine image) and the third image (L-spine image), with the same point selection process. For the aPTP approach, the implementation of an image stitching was performed based on the SIFT algorithm. The SIFT algorithm was used to detect the feature points created from an edge or corner of an object for vector calculation. The SIFT algorithm was applied in five steps; detection of the extrema in scale-space, keypoint localization, keypoint orientation assignment, Generation of keypoint descriptor and Point matching process [10].

First, the filterling process was applied to the process of extrema detection in scale-space to identify locations and scales that are invariant from different views of the same object. A scale-space of an image was generated by convolving the image with variable scale Gaussian kernel function.

L(x,y,σ)=G(x,y,σ)*I(x,y) (1)

$$G(x,y,\sigma )=\frac{1}{2\pi \sigma^{2}}e^{-(x^{2}+y^{2})/2\sigma^{2}}$$ (2)

Where *G*(*x*,*y*,*σ*) is the Gaussian function, *I*(*x*,*y*) is the object image, *x*,*y* is the coordinate location and * is the convolution operator.

The calculation of Difference of Gaussian (DoG), which can be used to detect the stable keypoint in the scale-space, was acquired from the difference between one scale and *k* times scale images.

D(x,y,σ)=(G(x,y,kσ)−G(x,y,σ))*I(x,y)=L(x,y,kσ)−L(x,y,σ) (3)

Where *D*(*x*,*y*,*σ*) is the Difference of Gaussian (DOG) and *k* is the scale factor. Local extrema of DOG images are calculated by comparing each sample point to its 8 neighbors at the same scale, and its 9 neighbors up and down scale. If calculated value is indicated the maximum or minimum, this location point is considered as an extremum.

Second, the keypoint localization process is applied to eliminate unnecessary points from the list of keypoints by finding those that have low contrast or poorly localized on edges. This process is done by using second-order Taylor expansion of the DoG function [11].

$$D=D+\frac{\partial {\text{D}}^{\text{T}}}{\partial \text{X}}\text{X}+\frac{1}{2}{\text{X}}^{\text{T}}\frac{\partial^{2}\text{D}}{\partial {\text{X}}^{2}}\text{X}$$ (4)

where *D*(*x*,*y*,*σ*) and its derivatives are evaluated at the sample point, and X = (*x*,*y*,*σ*)^T^ is the offset from this point [8]. The extremum location ($\hat{\text{X}}$) is determined by taking the derivative of this function with respect to X and setting it to zero [8].

$$\hat{\text{X}}=-{\left(\frac{\partial^{2}\text{D}}{\partial {\text{X}}^{2}}\right)}^{-1}\frac{\partial \text{D}}{\partial \text{X}}$$ (5)

In this paper, we used all absolute values for extremum less than 0.03, and we assumed image pixels were normalized, thus pixels ranges were from 0 to 1 [8]. If the function value is below a threshold value, then this point is excluded. This removes extrema that has low contrast. When the process of extrema elimination in the poor location was applied, it is important to consider a large principal curvature across the edge and a small one in perpendicular direction. To calculate the principle curvature, the ratios of Trace of Hessian matrix and Determinant were used. We assumed α is the eigenvalue with the largest magnitude and β is the smallest one. We calculated the sum of the eigenvalue from the trace of H and their product from the determinant as follows.

$$\text{Tr}\left(\text{H}\right)={\text{D}}_{\text{xx}}+{\text{D}}_{\text{yy}}=\alpha +\beta$$ (6)

$$\text{Det}(\text{H})={\text{D}}_{\text{xx}}{\text{D}}_{\text{yy}}-{\left({\text{D}}_{\text{yy}}\right)}^{2}=\alpha \beta$$ (7)

Using Eqs (6) and (7), we determined the ratio and we newly defined γ is the ratio between the α and β.

$$\frac{\text{Tr}{\left(\text{H}\right)}^{2}}{\text{Det}(\text{H})}=\frac{{\left(\alpha +\beta \right)}^{2}}{\alpha \beta }=\frac{{\left(\gamma +1\right)}^{2}}{\gamma }$$ (8)

Third, we computed the local gradient magnitude and orientation to assign a consistent orientation to the keypoints. The equations are as follows.

$$m(x,y)=\sqrt{(L(x+1,y)-L(x-1),y){)}^{2}+{\left(L(x,y+1)-L(x,y-1)\right)}^{2}}$$ (9)

$$\theta (x,y)=tan^{-1}((L(x,y+1)-L(x,y-1))/(L(x+1,y)-L(x-1,y))$$ (10)

where *m*(*x*,*y*) is the gradient magnitude, *θ*(*x*,*y*) is the orientation and *L*(*x*,*y*) is each Gaussian smoothed image. We created histogram from the gradient orientation of sample points within a 3∙σ window around the keypoint. Each sample added to the histogram is weighted with Gaussian window of 1.5∙σ. And then, we found the highest peak in the histogram and any other local peak within 80% of the height of the highest peak in order to create a keypoint.

Fourth, to compute the feature descriptor, the coordinate and gradient information are rotated to the keypoint orientation and then weighted by Gaussian function with 1.5∙σ. We used a set of 16 histograms, and these were aligned in a 4x4 grid, each with 8 orientation bins. Thus, we got the 128 elements feature vector for each keypoint.

Finally, the image matching process between the extracted SIFT feature points from a reference image and another image was applied using the calculation of Euclidean distance.

$$dis=\sqrt{{\left(x_{1}-x_{2}\right)}^{2}+{\left(y_{1}-y_{2}\right)}^{2}}$$ (11)

In the case of multi-slice images, the point selection is only performed at the most central slice among them, and other slices are combined with the selected point at the most central slice to stitch images. All of image processing was done in Matlab 7.8 (Mathworks, Natick, MA, USA) with a 2.6 GHz Intel Core 2 duo processor with 4 GB RAM. The GUI implementation is also performed by Matlab software.

For quantitative comparisons between the stitched images using SIFT algorithm and commercially mounted stitching algorithm in MRI system, we performed Bland-Altman analysis [12], and calculated Normalized Mean Square Error (NMSE) and Pearson’s correlation coefficients. These quantitative analysis procedures performed with images from 3.0 T MRI system (In 1.5 T MRI system, the stitching algorithms has not been mounted yet). Note that there is no gold standard or ground truth for stitching image results, we assumed that the stitched images from commercial MRI system as a reference images for quantitative analysis in this study.

## Results

**Fig 1:**
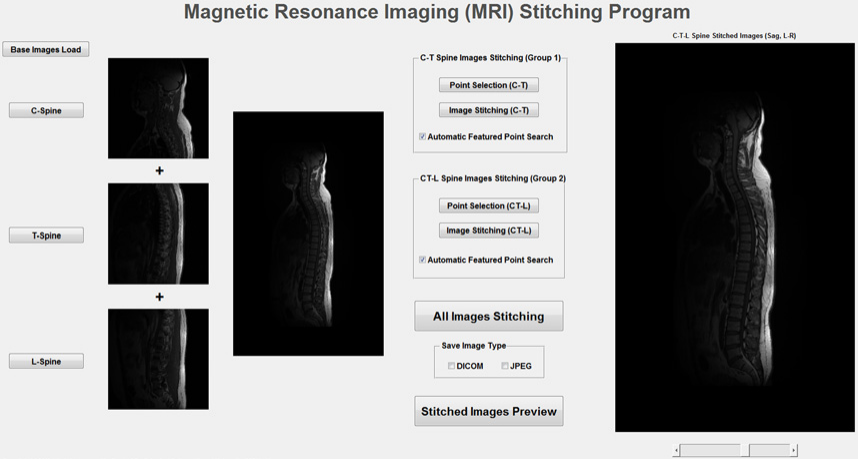
Developed GUI configuration program for C-T-L spine MRI stitching.

Developed GUI configuration for C-T-L spine MR images stitching program and its process is shown in Fig 1. It is the user’s choice to select mPTP or aPTP method. The image preview was provided in order to know and confirm each step of stitching process intuitively. It is possible to confirm the different cross-sectional multi-slice images using a scroll bar of the final image display. The stitched image results were saved in DICOM or JPEG image files. In the case of multi-slice images, the point selection was performed at the most central slice among them, and other slices were automatically stitched based on the selected points from the central slice.

**Fig 2:**
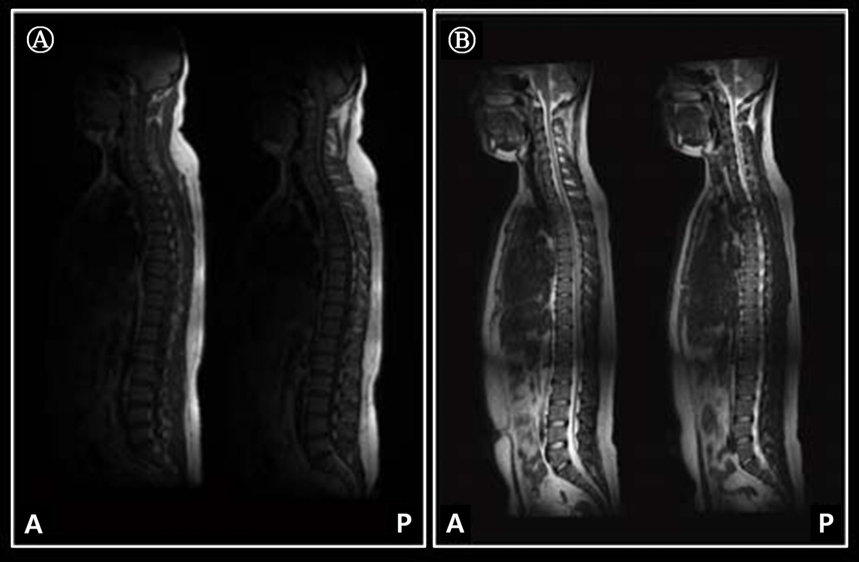
C-T-L spine MRI stitching result images (sagittal sections) using SIFT algorithm and developed GUI configuration for a representative subject. Datasets are acquired from 1.5 T MRI system (A) and 3.0 T MRI system (B).

Fig 2 shows the stitched C-T-L images for 3rd and 4th slices from representative subjects using developed GUI configuration in two different MRI systems: (A)- 1.5 T and (B)- 3.0 T MRI system.

**Fig 3:**
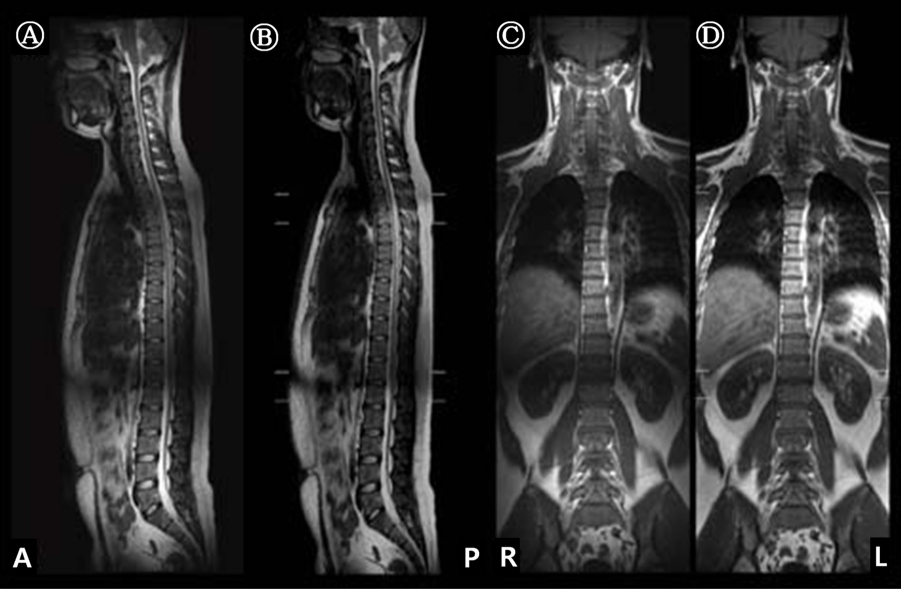
A representative subject’s stitching results for the use of SIFT algorithm on GUI configuration (A; sagittal section and C; coronal section) and the use of stitching algorithm commercially mounted on the MRI system (B; sagittal section and D; coronal section). All images acquired from 3.0 T MRI system.

Fig 3 shows the comparative results, which are sagittal and coronal section images from representative subject, between the use of our proposed stitching approach (SIFT) on GUI configuration (A and C) and the use of stitching algorithm commercially mounted on the 3.0 T MRI system (B and D). The calculated NMSE values from all subjects are represented 1.74 × 10^−4^ ± 3.68 × 10^−5^ (sagittal) and 1.85 × 10^−4^ ± 2.92 × 10^−5^ (coronal). For statistical results performed with paired t-test between the use of the SIFT algorithm and commercially mounted stitching algorithm, note that there is no significant differences in sagittal or coronal image sections. (all *p* > 0.05).

**Fig 4:**
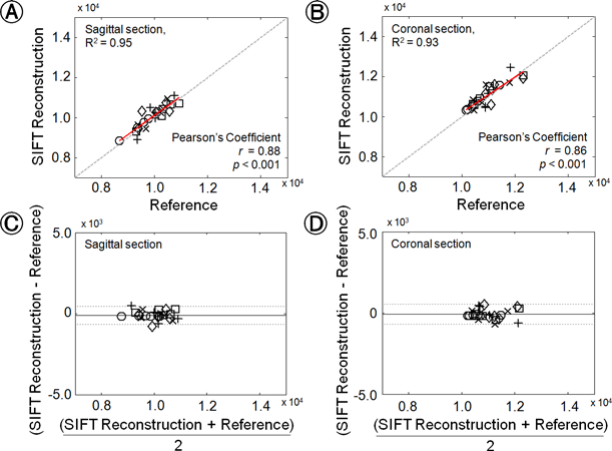
Quantitative comparison of signal intensities between the results using SIFT algorithm and commercially mounted stitching algorithm at 3.0 T MRI system (as reference image) for sagittal section (A) and coronal section (B). The red sold line represents the correlations and the linear regression of all values. The Pearson’s coefficient (*r*) and *p* values are provided for each image sections. (C) and (D) indicate the Bland-Altman plots of signal intensities acquired from stitching results using SIFT algorithm, compared with those by commercial mounted stitching algorithm for sagittal and coronal sections, respectively. The black solid line is the mean of the difference and the dotted lines bracket ±1.96 times the standard deviations of the means.

Fig 4 shows the quantitative comparison of the mean signal intensities from whole stitched images reconstructed with SIFT and commercially mounted algorithms in each subject’s image slices (A and B), and Bland-Altman analysis results (C and D). Notably, the calculated Pearson's coefficient values (*r*) represent high correlations between two stitched results performed with SIFT algorithm and commercially mounted stitching algorithm (*r* = 0.88; *p* < 0.001 for sagittal section; *r* = 0.86; *p* < 0.001 for coronal section). In addition, the signal intensities comparison results, which are located close to the y = x line, and high coefficients of determination values (R^2^ = 0.95 for sagittal section; R^2^ = 0.93 for coronal section) also clearly indicate that the feasibility and reliability for the use of SIFT algorithm to spine MR image stitching process. For the Bland-Altman analysis results, the mean differences and standard deviations are -104.48 ± 280.88 (Arbitrary Unit; A.U.), and -187.79 ± 315.78 A.U. for sagittal and coronal section results, respectively. The all differences between SIFT algorithm and commercially mounted stitching algorithm are within the ranges of ±1.96 times the standard deviations of the means, and there are no significant biases at sagittal and coronal section stitching results.

Furthermore, the computational time consumption of the overall imaging processes were 1.83 sec for 1.5 T MR spine images and 2.76 sec for 3.0 T MR spine images, providing to be highly correlated with the involved image matrix size.

## Discussion

Medical images generally have many different feature points. However, all of those points are not useful for image registration and segmentation processes. Thus, it is necessary to detect more accurate and important feature points of the images that are informative in one side and in the other side of target images for reducing the complexity. Especially, the SIFT algorithm is given a high degree of accuracy in the results as compared to another feature extraction methods. This advantage of SIFT algorithm is to be extended to more areas. In the computer vision and pattern recognition researches, the automatic detection of bilateral symmetry is a challenging task and it is currently have been overcome by the application of SIFT to detect the bilateral symmetry in object images [13, 14]. Moreover, in the previous studies, there were only a few researches on the applications of the SIFT algorithm for medical imaging field to track the signal features and to evaluate them for their research purpose such as image registration [10, 15, 16], motion detection [17, 18], and radiation therapy planning [19]. As a similar purpose of previous studies, we wanted to present a SIFT application to another medical imaging field, and confirm the possibility of our application.

Therefore, in this study, we proposed a C-T-L spine MR images stitching process based on the SIFT algorithm on GUI configuration for convenience of user interface and implementation. This implemented GUI software is worked by the user of stitching method (mPTP or aPTP) selection. We validated the accuracy and robustness of the proposed method through experiments and quantitative analysis. Based on our results, the stitched images using SIFT algorithm showed fine and reliable results, and also clearly showed the reproducibility of the use of SIFT algorithm compared with commercially mounted stitching algorithm on MRI system. In addition, to the best our knowledge, only one study was performed for the application of SIFT algorithm to the MR spine images stitching [20]. The previous research showed their stitched spine results without any quantifications and comparisons with other results, and we considered that these are the main drawback of the previous research. In this study, we also have applied the SIFT method as in previous study; however, unlike previous research, we have quantitatively evaluated our results compared with the results from commercially mounted algorithm in MRI system. Therefore, our studies objectively indicated the feasibility and possibility of the SIFT application to MR spine images stitching.

There are some limitations in this study. The application of SIFT algorithm has a possibility of mismatching between features, and it can lead to unexpected results in the matching procedures. Because we did not deal with this issue for our application, it can be a technical limitation in this study. Since the SIFT algorithm is widely used in various fields, this mismatching issue is sometimes considered as a weak-point of SIFT algorithm. Although all the quantitative and qualitative results showed good agreements with the results that derived from commercial MRI system, strictly speaking, it is hard to completely ignore the possibility of mismatching problem based on our results. To solve the mismatching problem technically, some methods have been introduced such as; random sample consensus (RANSAC) algorithm, which is an iterative parameter estimation method between inliers and outliers of data [21], and second-order Gaussian (SOG) algorithm, which gives an weighting to the matching points using Gaussian function depending on the Euclidean distance to adjust the matching points in threshold values [22]. Therefore, in order to pursue higher accuracy of the stitching results, we will try SIFT combined with above mentioned algorithms to eliminate the mismatching problems in the future. Moreover, our proposed approach relies on the early stage of SIFT algorithm to MR spine image stitching application, so further study will be performed on a large number of subjects to improve the performance. Finally, this approach is processed in single GPU system. In the future study, the efficiency of computation time consumption of the feature extraction will be improved to use multi GPU-based parallel computation and multi-core processing implementation.

## Conclusions

We provided preliminary findings of the feasibility and the validity of the SIFT algorithm application. As shown in the results, the algorithm could be performed well in the MR spine images stitching process. We believe that our approach could be extended to the other image stitching fields such as whole-body MRIs and other imaging modalities as well, and could be helpful for clinical applications and diagnosis improvements.
